# Supplementary material for: Laparoscopy-assisted percutaneous correction of abdominal wall defects in the umbilical region in a cadaveric model of bovine fetus
Source: PLoS One. 2023 May 31;18(5):e0285988. doi: 10.1371/journal.pone.0285988 (PMC10231838; doi:10.1371/journal.pone.0285988)
Supplement: S1 File — (DOCX) [file pone.0285988.s001.docx]

**Highlights**

- Umbilical abdominorrhaphy by ventral laparotomy provided access and ensured exposure of the abdominal cavity and umbilical remnants
- Bovine fetuses (corpses) are efficient to create a study model with the realization of the defect in the abdominal wall
- Laparoscopy allows for a wider diagnostic exploration of the abdominal cavity and umbilical remnants
- The laparoscopic technique allows the creation of a study model for video-assisted percutaneous suturing with two portals
- Percutaneous suturing using a guiding catheter is effective for reducing periumbilical abdominal wall injury
