# Supplementary material for: Laparoscopy-assisted percutaneous correction of abdominal wall defects in the umbilical region in a cadaveric model of bovine fetus
Source: PLoS One. 2023 May 31;18(5):e0285988. doi: 10.1371/journal.pone.0285988 (PMC10231838; doi:10.1371/journal.pone.0285988)
Supplement: Attachment — Submitted filename: Response to reviewers R2.docx [file pone.0285988.s002.docx]

**Ref:** PONE-D-22-23559R1

Laparoscopy-assisted percutaneous correction of abdominal wall defects in the umbilical region in an animal model (study model)

**Answers to the reviewers**

**Academic Editor**

English-language proofreading of the new version.

**Response:** Yes (Enclosed English proofreading certificate).

- Keywords: please replace “invasive minimally technique” with “minimally invasive technique”

**Response:** Suggestion inserted in the manuscript.

-Title: please avoid redundancy in the title; First suggested option for title: “Laparoscopy-assisted percutaneous correction of abdominal wall defects in the umbilical region in an animal model”. Second suggested option for title: “Laparoscopy-assisted percutaneous correction of abdominal wall defects in the umbilical region in a cadaveric model of bovine fetus”.

**Response:** Second suggestion inserted in the manuscript.

-lines 37-42: please rephrase to facilitate the reader's understanding.

**Response:** Suggestion inserted in the manuscript.

“The procedures were performed in two steps. The first step consisted of creating an abdominal wall defect in the umbilical region by laparoscopic approach in an iatrogenic manner (Step 1: E1). The second stage consisted of conventional abdominorrhaphy of the umbilical region wall defect in the CG group and video-assisted percutaneous suturing of the edges of the iatrogenic abdominal wall defect in the VG group, until reversal of the laparoscopic accesses (Step 2: E2).”

-line 89: please replace “All these pregnant cows” with “All the pregnant cows”.

**Response:** Suggestion in manuscript.

-line 91: delete “without causing pain or suffering to the animals.” To avoid redundancy as cadavers are used.

**Response:** Suggestion inserted in manuscript.

-line 126: please replace "lighting" with "lighting cable".

**Response:** Correction inserted in manuscript.

-lines 164-169 (capture of figure 4): please replace “nylon” with “nylon thread”, as in caption of figure 5.

**Response:** Suggestion inserted in manuscript.

-line 187: please replace “, surgical techniques:” with “;”

**Response:** Substitution realized in manuscript.

-lines 202-203: please rephrase to facilitate the reader's understanding.

**Response:** Correction inserted in manuscript.

“The right lateral approach, with the establishment of laparoscopic accesses, allowed the execution of the procedures properly, as it guaranteed access to the abdominal wall defect with a wide field of view, contributing to the feasibility of the technique.”

-line 212: Since in the table is reported the p-values, it is suggested to delete “p≤0.05” from the caption.

**Response:** Suggestion inserted in manuscript.

-line 222: It is not clear what these values “(08/01–12.5%)” indicates. Maybe you mean “(1 case out of 8, 12.5%)”? Please clarify or delete.

**Response:** Values excluded in the manuscript.

-lines 224-225: please rephrase to facilitate the reader's understanding.

**Response:** Correction inserted in manuscript.

“Dieresis of the musculature and peritoneum was performed to correct the iatrogenic defect of the abdominal wall with subsequent dermarrhaphy by separate simple suture in the CG group.”

-line 236: please delete “and other methods”.

**Response:** Suggestion inserted in manuscript.

-lines 271-273: please rephrase to facilitate the reader's understanding.

**Response:** Correction inserted in manuscript.

“The time for correction of the abdominal wall defect in the umbilical region was shorter with the aid of laparoscopy”
